# Fecal medicines used in traditional medical system of China: a systematic review of their names, original species, traditional uses, and modern investigations

**DOI:** 10.1186/s13020-019-0253-x

**Published:** 2019-09-13

**Authors:** Huan Du, Ting-ting Kuang, Shuang Qiu, Tong Xu, Chen-Lei Gang Huan, Gang Fan, Yi Zhang

**Affiliations:** 1 School of Pharmacy, Chengdu University of Traditional Chinese Medicine, Chengdu, 611137 China; 2 School of Ethnic Medicine, Chengdu University of Traditional Chinese Medicine, Chengdu, 611137 China; 3 School of Foreign Language, Chengdu University of Traditional Chinese Medicine, Chengdu, 611137 China

**Keywords:** Fecal medicines, Traditional Chinese medicine, Gut microbiota, Fecal microbiota transplantation, Gastrointestinal diseases

## Abstract

In China, the medical use of fecal matter (fresh fecal suspension or dry feces) can be dated back to the fourth century, approximately 1700 years ago. In long-term clinical practice, Chinese doctors have accumulated unique and invaluable medical experience in the use of fecal materials. In view of their good curative effect and medicinal potential, fecal medicines should be paid much attention. This study aimed to provide the first comprehensive data compilation of fecal medicines used in various Chinese traditional medical systems by bibliographic investigation of 31 medicine monographs and standards. A total of 54 fecal medicines were found to be used in 14 traditional Chinese medical systems. Their names, original species, medicinal forms, and traditional uses were described in detail. These fecal medicines were commonly used to treat gastrointestinal, nervous system, skin, and gynecological diseases. Commonly used fecal medicines include Wu-Ling-Zhi, Jiu-Fen and Hei-Bing-Pian. The information summarized in this study can provide a good reference for the development and utilization of fecal medicines. Further studies are necessary to prove their medicinal value, identify their active ingredients, and elucidate their mechanisms of action so that more people can accept these special medicines.

## Background

Traditional medicines have been used for prevention and treatment of diseases for thousands of years in China. In recent decades, they have attracted worldwide attention due to their reliable therapeutic efficacy and low side effects. During the long-term struggle against diseases, ancient Chinese doctors found that some unexpected materials, such as human or animal feces, could also effectively treat diseases. In China, the medical use of fecal matter (fresh fecal suspension or dry feces) has a long history. During the Eastern Jin dynasty (ad 300–400 years), “*Zhou Hou Bei Ji Fang*”, a well-known monograph of traditional Chinese medicine (TCM) written by Hong Ge, recorded a case of treating patients with food poisoning or severe diarrhea by ingesting human fecal suspension (known as yellow soup or Huang-Long decoction) [1]. During the Tang dynasty, Yutuo Ningma Yundan Gongbu compiled a world-famous book of Tibetan medicine called “*The Four Medical Tantras*”, which recorded that digestive diseases can be treated with the processed product of the feces of *Sus scrofa* (Hei-Bing-Pian in Chinese) [2]. Later, the “*Compendium of Materia Medica*” (a masterpiece of herbalism written by Shizhen Li during the Ming dynasty) described a series of prescriptions for treating diarrhea, rheumatism, jaundice, fever, and pain using fresh fecal suspension or dry feces [3]. In addition, “*Jing Zhu Materia Medica*” written by Danzeng Pengcuo Dimaer in the nineteenth century recorded that Hei-Bing-Pian and the dry feces of *Gypaetus barbatus* or *Aegypius monachus* (Jiu-Fen in Chinese) are commonly used to treat dyspepsia and gastric ulcer [4]. These records indicate that fecal medicines are widely used and occupy an important position in Chinese traditional medical systems.

In long-term clinical practice, Chinese doctors have accumulated unique experience in the use of fecal medicines. For example, the dry feces of *Trogopterus xanthipes* (Wu-Ling-Zhi in Chinese) is often used to treat blood stasis, swelling and aching due to traumatic injury [5]. Jiu-Fen is good at treating gastrointestinal diseases, such as dyspepsia, weak gastrointestinal function and gastric ulcer. Hei-Bing-Pian can treat diseases, such as indigestion, diarrhea and distending pain in the stomach [6]. These traditional medication experiences are undoubtedly valuable assets and can provide a reference for modern drug development. However, documents on the traditional use of fecal medicines are scattered and lack systematic collation.

In this review, we comprehensively collect and summarize the names, origins, and treated diseases of fecal medicines that have been used in some Chinese traditional medical systems, including TCM, Tibetan ethnic medicine (EM), Oroqen EM, Kazak EM, Uygur EM, Mongolian EM, Nu EM, Yao EM, Wa EM, Tujia EM, Korean EM, Jino EM, Hani EM, and Dai EM. In addition, we review the most frequently used fecal medicines in terms of their origins, traditional uses, chemical constituents, and pharmacological activities. Such information can provide a good reference for their development and utilization. These fecal medicines may be a valuable gift from Chinese traditional medicine to the world and has potential as drug candidates for the treatment of some chronic diseases, such as gastrointestinal diseases.

## Methods

We have manually searched 31 related medicine monographs and drug standards, such as “*Zhou Hou Bei Ji Fang*”, “*Compendium of Materia Medica*”, “*Jing Zhu Materia Medica*”, “*Dictionary of Chinese Ethnic Medicine*”, “*Drug Standards of Tibetan Medicine*”, “*Lan Liu Li*”, “*Pharmacopoeia of the People’s Republic of China”*, and “*Chinese Tibetan Materia Medica*”, to obtain the information about the names, origins, traditional uses, and treated diseases of fecal medicines. In addition, we have searched the online Chinese literature databases (i.e., Wan fang and CNKI) and international English databases (i.e., PubMed, ISI Web of Science and Google Scholar), using their vernacular or Latin names as keywords, to obtain their chemical constituents and biological effects.

## Results

This review recorded 54 fecal medicines that have been used in 14 Chinese traditional medical systems. Their names, original species, medicinal forms, and treated diseases are presented in Tables 1 and 2. These 54 medicines mainly originate from the feces of 56 animals. Among medicinal forms used, dry feces is the most frequently used (66.67%), followed by processed feces (29.63%) and fresh fecal juice (3.70%). In addition, we found that these 54 fecal medicines are mainly used to treat gastrointestinal (37.04%), nervous system (22.22%), skin (22.22%), ophthalmic (18.52%), and gynecological diseases (16.67%).

**Table 1:** Fecal medicines used in the traditional Chinese medicine (TCM) system

No.	Animal species	Medicinal form	Chinese name	Traditional uses (treated diseases)	Refs.
1	*Trogopterus xanthipes* Milne-Edwards	Dry feces	Wu-Ling-Zhi	Stabbing pain in chest and abdomen, dysmenorrhea, amenorrhea, swelling and aching due to traumatic injury, and snake bites	[5, 8, 52]
2	*Bombyx mori* L.	Dry feces	Can-Sha	Rheumatism, arthralgia, skin numbness, cold pain in waist and legs, rubella itching, and headache	[3, 52]
3	*Vespertilio superans* Thomas	Dry feces	Ye-Ming-Sha	Night blindness, swelling and pain in the eyes, infantile malnutrition, scrofula, and malaria	[5, 53]
	*Rhinolophus ferrumequinum* Schreber				
	*Plecotus auritus* L.				
	*Hipposideros armiger* Hodgson				
4	*Lepus mandschuricus* Radde	Dry feces	Wang-Yue-Sha	Blurred vision, hemorrhoids and fistula, and infantile malnutrition	[52, 54, 55]
	*Lepus sinensis* Gray				
	*Lepus oiostolus* Hodgson				
5	*Gallus gallus domesticus* Brisson	Dry feces	Ji-Shi-Bai	Jaundice, gonorrhea, wandering arthritis, tetanus, spasm of muscles and tendons, and diabetes	[3, 56]
6	*Rattus flavipectus* Milne-Edwards	Dry feces	Liang-Tou-Jian	Fever due to typhoid, abdominal pain, stranguria with turbid urine, amenorrhea, infantile malnutrition, mammary abscess, and furuncle	[56–58]
	*Rattus norvegicus* Berkenhout				
7	*Columba livia domestica* L.	Dry feces	Zuo-Pan-Long	Abdominal mass, scrofula and tetanus	[3, 57]
8	*Physeter macrocephalus* L.	Dry feces	Long-Xian-Xiang	Coma due to stuffiness, abdominal pain, cough and dyspnea, and gonorrhea	[52, 56]
9	*Passer montanus* L.	Dry feces	Bai-Ding-Xiang	Abdominal mass, carbuncle and furuncle, blurred vision, pterygium, and dental caries	[55, 56, 58]
10	Homo sapiens	Fresh fecal juice	Yellow soup or Huang-Long decoction	Food poisoning, severe diarrhea, heat toxin, and unconsciousness due to high fever	[1, 3]
11	*Hirundo daurica* L.	Dry feces	Yan-Zi-Fen	Edema, malaria, insect poison, ulcer and sore	[3, 56]
12	*Pavo muticus* L.	Dry feces	Kong-Que-Fen	Excessive leucorrhea in women, dysuria and furuncle	[3, 56]
13	*Bos taurus domesticus* Gmelin	Fresh fecal juice or dry feces	Niu-Fen	Fresh fecal juice can cure jaundice caused by diabetes, beriberi, cholera, and dysuria; dry feces can treat malignant sore, cervical lymph node and tuberculosis fistula	[3, 56]
	*Bubalus bubalis* L.				
14	*Ochotona thibetana* Milne-Edwards	Dry feces	Cao-Ling-Zhi	Irregular menstruation, stagnant abdominal pain, stomach pain, traumatic injury, and blood stasis	[56, 57]
	*Ochotona erythrotis* Buchner

**Table 2:** Fecal medicines used in other traditional ethnic medicine (EM) systems in China

No.	Animal species	Medicinal form	Traditional medical systems	Traditional uses (treated diseases)	Refs.
1	*Gypaetus barbatus* L.	Dry feces	Tibetan EM and mongolian EM	Dyspepsia, abdominal distension, intestinal tumor, gastric ulcer, weak gastrointestinal function, sores and carbuncles	[2, 4, 6]
	*Aegypius monachus* L.				
2	*Sus scrofa* L.	Processed feces	Tibetan EM and mongolian EM	Dyspepsia, biliary diseases, plague and distending pain of stomach	[6, 36, 59]
3	*Sus scrofa domestica* Brisson	Processed feces	Tibetan EM	Dyspepsia, plague and biliary tumor	[4, 36, 60]
4	*Petaurista xanthotis* Milne-Edwards	Dry feces	Tibetan EM	Stomach pain, amenorrhea and dysmenorrhea	[59, 60]
5	*Pteromys volans* L.	Dry feces	Oroqen EM	Dysentery and diarrhea	[60]
			Kazak EM	Metrorrhagia, amenorrhea, and snake bites (external use)	[60]
			Uygur EM	Eczema, itching, amenorrhea, dysmenorrhea, stomach pain, and traumatic injury	[61]
6	*Riparia riparia* L.	Processed feces	Tibetan EM	Bloody dysentery, chronic diarrhea, women amenorrhea, and hematuria	[59, 60]
7	*Felis ocreata domestica* Brisson	Processed feces	Tibetan EM	Manic psychosis or madness	[36, 60]
8	*Upupa epops* L.	Processed feces	Tibetan EM	Psychopathy	[4, 59]
9	*Vulpes vulpes* L.	Dry feces	Tibetan EM	Psychopathy and epilepsy	[4, 60]
10	*Trogopterus xanthipes* Milne-Edwards	Dry feces	Mongolian EM	Diarrhea, gout and itching	[62]
			Nu EM	Cold, whooping cough and fever	[60]
			Tibetan EM	Stomach pain, dysmenorrhea and amenorrhea	[59, 60]
			Tujia EM	Blood stasis, furuncles, traumatic injury, dysmenorrhea, and snake bites (external use)	[60]
			Korean EM	Stabbing pain in chest and abdomen, dysmenorrhea, amenorrhea, swelling and aching due to traumatic injury	[60]
			Dai EM	Amenorrhea, dysmenorrhea, pain due to blood stasis, and snake bites (external use)	[60]
			Yao EM	Dysmenorrhea, amenorrhea, and epilepsy	[63]
11	*Pipistrellus abramus* Temminck	Dry feces	Wa EM	Asthma, burn, night blindness, and infantile malnutrition	[60]
			Tibetan EM	Epilepsy	[4, 60]
			Tujia EM	Night blindness, cataract, infantile malnutrition, and corneal nebula	[64]
12	*Vespertilio superans* Thomas	Dry feces	Korean EM	Night blindness, intermittent fever, cataract, and underarm odor	[60]
			Yao EM	Night blindness, corneal nebula, infantile malnutrition, and scrofula	[63]
13	*Plecotus kozlovi* Bobrinski	Processed feces	Tibetan EM	Eye diseases, scrofula and infantile malnutrition	[60]
14	*Myotis mystacinus* Kuhl	Processed feces	Tibetan EM	Psychosis and epilepsy	[60]
15	*Lepus capensis* L.	Dry feces	Tibetan EM	Blurred vision, hemorrhoids and fistula, and infantile malnutrition	[4, 59]
16	*Equus caballus orientalis* Noack	Processed feces	Tibetan EM	Various parasitic diseases and vomiting	[65, 66]
17	*Gallus gallus domesticus* Brisson	Dry feces	Korean EM	Jaundice, gonorrhea, tetanus, and diabetes	[60]
			Tibetan EM	Eye diseases	[4, 60]
			Dai EM	Shoulder arthritis, tetanus and corneal scar	[60]
18	*Rattus rattus* L.	Dry feces	Tibetan EM	Epilepsy	[59, 60]
19	*Physeter macrocephalus* L.	Dry feces	Uygur EM	Neurasthenia, memory loss and psychological impotence	[60]
20	*Passer montanus* L.	Dry feces	Jino EM	Manic psychosis or madness	[60]
			Hani EM	Hernia	
			Tibetan EM	Sores and furuncles (external use)	[59, 60]
21	*Columba rupestris* Pallas	Dry feces	Tibetan EM	Swelling and suppuration	[2, 60]
22	*Pavo muticus* L.	Processed feces	Tibetan EM	Inflammation	[4, 36, 59]
23	*Bos taurus domesticus* Gmelin	Processed feces	Tibetan EM	Food poisoning, limbs pain, and spasm	[4, 36, 59]
24	*Canis lupus* L.	Processed feces	Tibetan EM and mongolian EM	Psychopathy and swelling	[4, 59]
25	*Tetraogallus tibetanus* Gould	Dry feces	Tibetan EM	Various swelling	[2, 36]
26	*Bubo bubo hemachalana* Hume	Processed feces	Tibetan EM	Psychopathy and epilepsy	[4, 59, 67]
27	*Pica pica* L.	Dry feces	Tibetan EM	Skin diseases, such as sores and furuncles	[59, 60]
28	*Streptopelia orientalis* Latham	Dry feces	Uygur EM	Purulent secretion of the ear; Pain caused by ear diseases	[61]
29	*Corvus corax* L.	Processed feces	Tibetan EM	Bromhidrosis, epilepsy, cough, and psychopathy	[59, 60]
30	*Canis lupus familiaris* L.	Processed feces	Tibetan EM	Psychopathy and swelling; Syphilis, psoriasis and anthracnose (external use)	[4, 59, 60]
31	*Elephas maximus* L.	Dry feces	Dai EM	Ophthalmitis	[60]
32	*Equus asinus* L.	Processed feces	Tibetan EM	Sores and furuncles (external use) and rabies	[4, 59]
33	*Phalacrocorax carbo* L.	Dry feces	Korean EM	Pigmented naevus (external use)	[60]
34	*Buteo hemilasius* Temminck et Schlegel	Dry feces	Tibetan EM	Sores and furuncles (external use)	[4, 59]
35	*Macaca mulatta* Zimmermann	Dry feces	Tibetan EM	Inflammation, swelling and dysentery	[59, 60]
36	*Ovis aries* L.	Dry feces	Tibetan EM	“Huang-Shui” disease in arms and legs (external fumigation)	[59, 60]
37	*Capra hircus* L.	Dry feces	Tibetan EM	Heat toxin syndrome, nervous system diseases and leprosy	[4, 60]
			Korean EM	Child dysentery, borborygmus and convulsive epilepsy	[60]
38	*Tetrao urogalloides* Middendorf	Dry feces	Tibetan EM	Psychopathy and swelling	[4]
39	*Lutra lutra* L.	Processed feces	Tibetan EM	Uterine diseases	[4, 60]
40	*Moschus sifanicus* Przewalski	Dry feces	Tibetan EM	Limb numbness, paralysis and blood stasis	[4, 36, 59]

### Fecal medicines used in the TCM system

Traditional Chinese medicine is the most representative traditional medical system in China. It has a long history of more than 2500 years. In recent decades, TCM has attracted global attention due to its reliable therapeutic efficacy. Generally, TCM uses herbs, animals or minerals to treat diseases. In long-term clinical practices, animal feces have been found to be effective in treating some specific diseases under the guidance of TCM theory. As early as the Eastern Jin dynasty, human fecal juice (i.e., yellow soup) has been used by TCM practitioners to treat severe diarrhea [1]. At present, some fecal medicines are still used in the clinical practice of TCM. In the 2015 edition *of Chinese Pharmacopoeia* [7], 18 preparations have been found to contain fecal medicines (Table 5). For example, “Shi-Xiang-Zhi-Tong Powder” and “Tong-Jing Pills” contain Wu-Ling-Zhi, and “Huang-Lian-Yang-Gan Pills” contains Ye-Ming-Sha (dry feces of some kinds of bats).

**Fig. 1:**
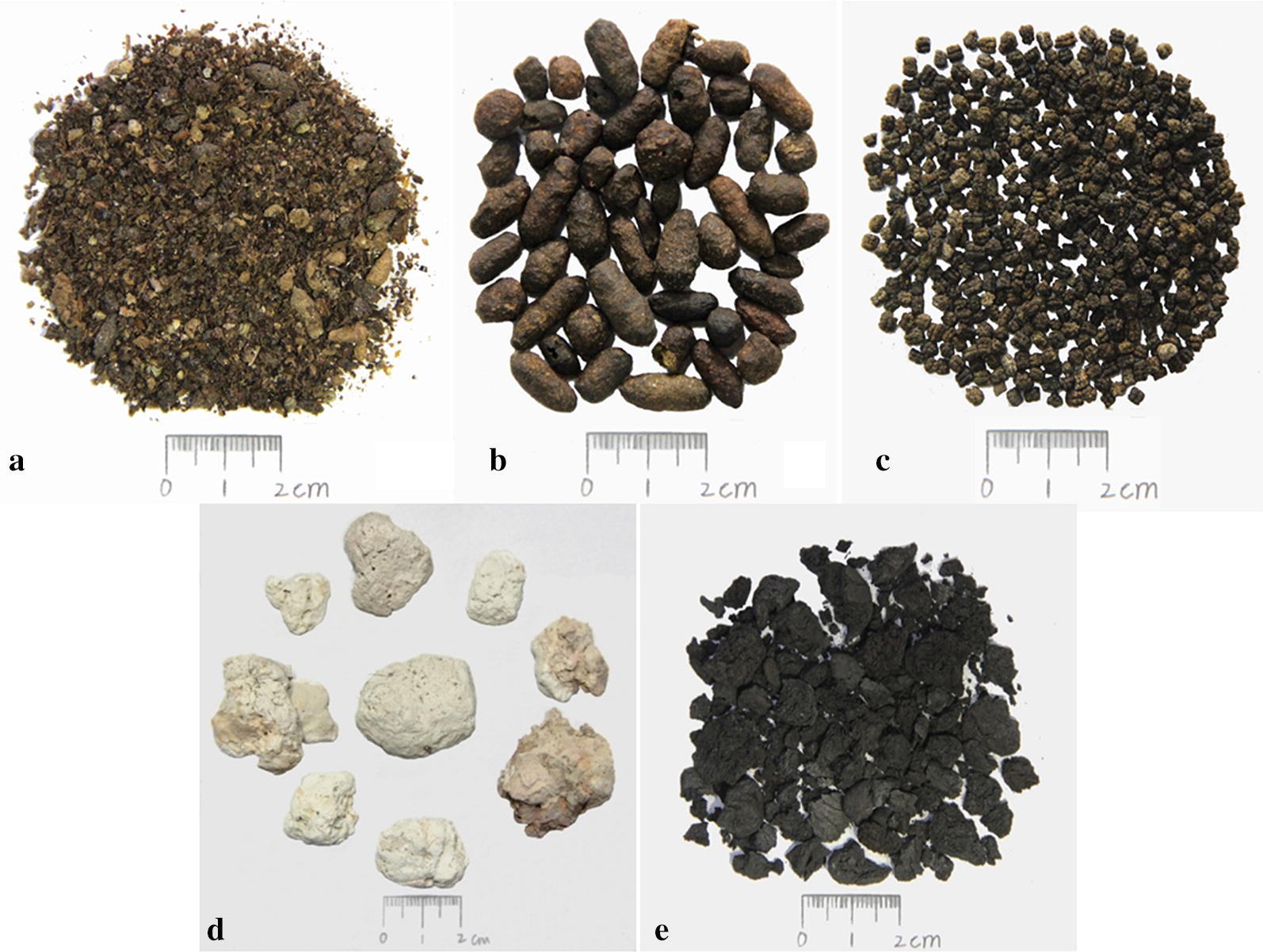
The commonly used fecal medicines in traditional medical system of China (**a** Ye-Ming-Sha; **b** Wu-Ling-Zhi; **c** Can-Sha; **d** Jiu-Fen; **e** Hei-Bing-Pian)

In the present study, a bibliographic investigation of TCM monographs and drug standards revealed 14 kinds of fecal medicines that are commonly used in the TCM system. They mainly come from the feces of 22 animals and are widely used to treat dysmenorrhea, amenorrhea, abdominal mass, diarrhea, and blurred vision. Additional information on these 14 medicines is provided in Table 1. Wu-Ling-Zhi is the most representative fecal medicine in the TCM system (Fig. 1). Therefore, its traditional uses, chemical constituents and pharmacological activities are described in detail in the subsequent sections.

#### The dry feces of *Trogopterus xanthipes* (Wu-Ling-Zhi in Chinese)

Wu-Ling-Zhi (Fig. 1b), also named as Goreishi or Trogopterorum faeces, is one of the commonly used fecal medicines. It derives from the dry feces of *Trogopterus xanthipes*. Wu-Ling-Zhi was first recorded in the classic Chinese medicinal book “*Kai Bao Ben Cao*” compiled in the Song Dynasty [8]. Its traditional uses were described in several TCM monographs and drug standards. For example, “*Ben Cao Jing Shu*” recorded that Wu-Ling-Zhi had a good therapeutic effect on stabbing pain caused by blood stasis [9]. In addition, the *Chinese Pharmacopoeia* 1990 edition recorded that Wu-Ling-Zhi had good effects of promoting blood circulation, removing blood stasis and relieving pain, and was usually used to treat the stabbing pain in the chest and hypochondrium, dysmenorrhea, amenorrhea, swelling and aching due to traumatic injury, postpartum blood stasis, and snake bites.

**Fig. 2:**
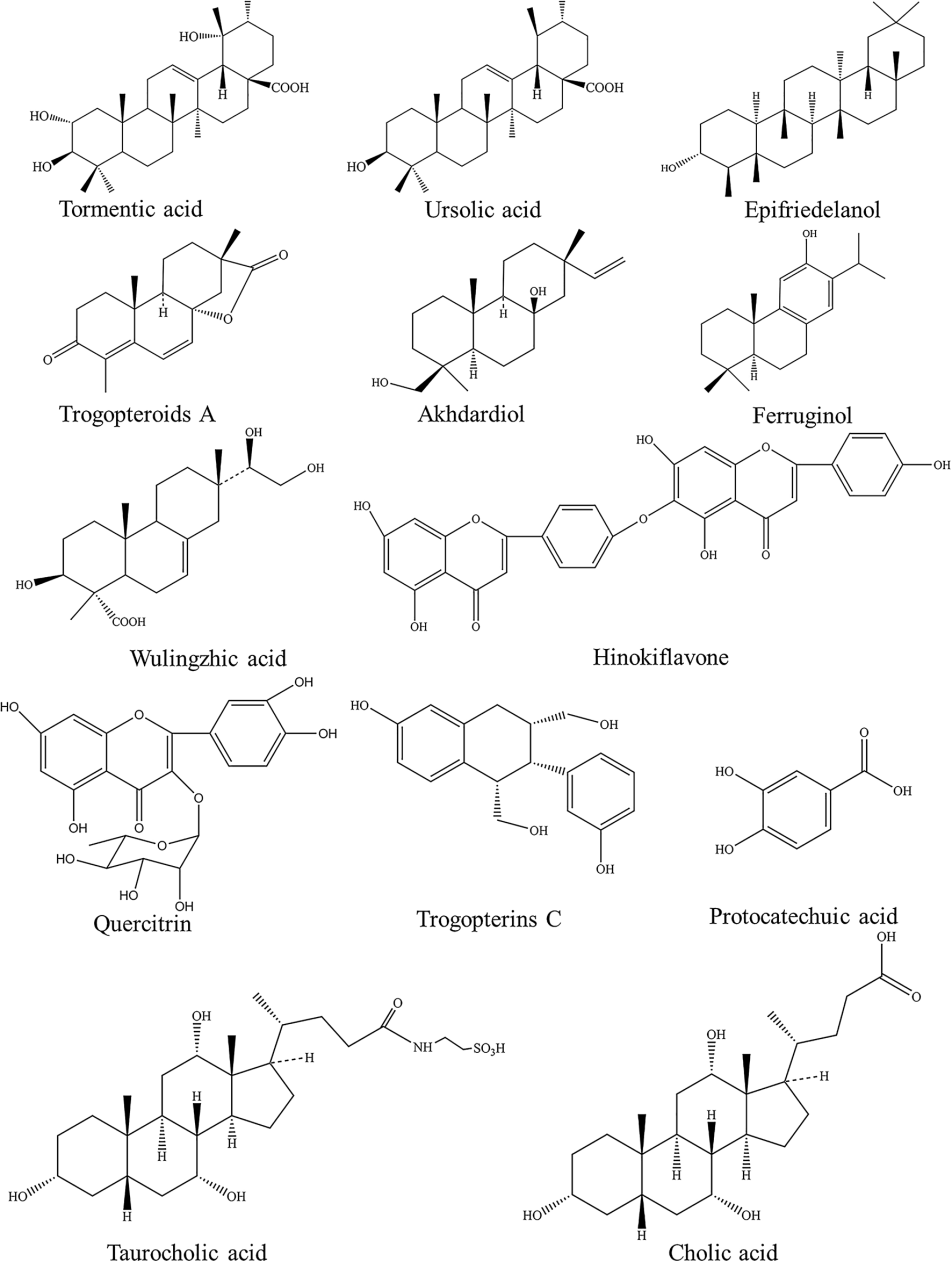
Chemical structures of representative compounds isolated from Wu-Ling-Zhi and Hei-Bing-Pian

So far, some chemical constituents categorized as terpenoids, flavonoids, lignans, sterols, and esters have been isolated from Wu-ling-zhi. The chemical structures of representative compounds are shown in Fig. 2. Numata et al. [10] found that the feces of *T. xanthipes* contain several cytotoxic triterpenes, namely, pomolic acid, 3-*O*-*cis*-*p*-coumaroyltormentic acid, 2α-hydroxyursolic acid, and jacoumaric acid. Subsequently, they isolated three new ursane-type triterpenes (i.e., goreishic acids I, II and III) from the feces of *T. xanthipes* in 1990 [11]. In addition, 19 diterpenoids including three new isopimarane diterpenoids (trogopteroids A–C) and four new aromatic diterpenoids (trogopteroids D–G) were isolated from the feces of *T. xanthipes* [12]. Yang et al. [13] also isolated two new diterpenoids (wulingzhic acid A and wulingzhic acid B) from the feces of *T. xanthipes*. Additionally, the isolation and structural elucidation of flavonoids in Wu-Ling-Zhi were done by Yang et al. [14]. Seven flavonoids such as kaempferol 3-*O*-α-l-(4″E-*p*-coumaroyl)-rhamnoside, hinokiflavone, afzelin, and quercitrin were found. In 2012, four new neolignans were obtained from the ethyl acetate fraction of methanol extract of Wu-Ling-Zhi [15]. Subsequently, they also isolated neolignans that had been reported before from its methanolic extract and named trogopterins A–C [16]. Moreover, Yang et al. [17] isolated four new fatty acid esters from the feces of *T. xanthipes*. Currently, it was reported that dihydrositosterol, epifriedelanol, 5-methoxy-7-hydroxycoumarin, β-sitosterol, ursolic acid, protocatechuic acid, and daucosterol were also isolated from Wu-Ling-Zhi [18, 19]. Moreover, some volatile compounds identified by capillary gas chromatography combined mass spectrometry were also found in Wu-ling-zhi, such as dodecanoic acid, alpha-cedrol, tetradecanoic acid, and benzaldehyde [20].

At present, there are some studies involving the quality control of Wu-Ling-Zhi. Yerigui et al. [21] quantified five bile acids (i.e., cholic acid, deoxycholic acid, lithocholic acid, ursodeoxycholic acid, and taurocholic acid) in Wu-Ling-Zhi by using ultra high-performance liquid chromatography-mass spectrometry. Jiao et al. [22] established a thin layer chromatography (TLC) method for qualitative identification of Wu-Ling-Zhi, and developed a high performance liquid chromatography (HPLC) method to simultaneously quantify three active ingredients (protocatechuic acid, hinokiflavone and amentoflavone) in Wu-Ling-Zhi. Recently, Chen et al. also established the quality standard of Wu-Ling-Zhi. They qualitatively and quantitatively analyzed quercetin, kaempferol and amentoflavone in Wu-Ling-Zhi by TLC and HPLC, respectively [23]. These results can provide important reference for the quality control of Wu-Ling-Zhi.

Moreover, it is worth pointing out that extracts or chemical constituents obtained from the Wu-Ling-Zhi have been proved to possess a wide spectrum of pharmacological activities, such as anti-inflammatory, anti-cerebral ischemia, anti-gastric ulcer, and antithrombin effects. The basic pharmacological data of Wu-Ling-Zhi extracts and some isolated compounds are shown in Tables 3 and 4. Kim et al. [24] reported that Wu-Ling-Zhi extract could reduce lipopolysaccharide-induced NO and cytokines production. Wang et al. [25] found that the ethyl acetate extract of Wu-Ling-Zhi showed obvious inhibitory effects on xylene-induced ear swelling in mice and carrageenan-induced paw swelling in rats (400 mg/kg, ip), and it could also significantly inhibit the proliferation of granulation tissue in mice (800 mg/kg, ip). These findings indicated that Wu-Ling-Zhi has obvious anti-inflammatory effect. Furthermore, the ethyl acetate extract of Wu-Ling-Zhi was also found to be able to protect gastric mucosa and prevent experimental gastric ulcer by inhibiting gastric acid secretion [26]. It was reported that the aqueous extract of Wu-Ling-Zhi could significantly prolong the survival time of mice with incomplete cerebral ischemia, reduce the brain water content, brain index and malondialdehyde (MDA) level, and increase superoxide dismutase (SOD) activity in rats, indicating that Wu-Ling-Zhi has good protective effect against cerebral ischemia [27]. Moreover, the aqueous extract of Wu-Ling-Zhi could down-regulate the expression of intercellular adhesion molecule-1 in experimental atherosclerotic rats and reduce the degree of vascular endothelial lesions, which may account for the anti-arteriosclerosis inflammatory effects of Wu-Ling-Zhi [28]. Currently, several compounds isolated from Wu-Ling-Zhi, such as 3-*O*-α-l-(2″*E*,4″*E*-di-*p*-coumaroyl)-rhamnoside, bis(7-hydroxyheptyl) decanedioate and bis(7-hydroxyheptyl) octanedioate, were found to have significant antithrombin activity [14, 17]. Besides, a recent study showed that Wu-Ling-Zhi could trigger caspase dependent apoptosis in breast cancer cells (MCF-7 cells) [29].

**Table 3:** Pharmacological activities and mechanisms of some compounds isolated from Wu-Ling-Zhi and Hei-Bing-Pian

Name	Classification	Compound	Pharmacological activity	Effect and mechanism	Refs.
Wu-Ling-Zhi	Terpenoids	Tormentic acid	Antiangiogenic activity	Controlling abnormal proliferation and cell death resistance of vascular smooth muscle cell without affecting the normal vasculature	[68]
		Euscaphic acid	Anti-inflammatory activity	Inhibition of LPS-induced inflammatory responses by interference with the clustering of TRAF6 with IRAK1 and TAK1	[69]
		Jacoumaric acid	Cytotoxic activity	Significant cytotoxicity effect against P-388 lymphocytic leukemia cell	[10]
		3-*O*-*cis*-*p*-coumaroy ltormentic acid			
		2α-hydroxy ursolic acid	Anticancer activity	Inhibition of cell proliferation and induction of apoptosis by regulating the p38/MAPK signal transduction pathway	[70]
		Pomolic acid	Anti-inflammatory and apoptotic activities	Inhibiting inflammatory response by regulating human neutrophil function	[71]
		Ursolic acid	Anticancer and anti-inflammatory activities	Inhibition of tumor growth and induction of apoptosis by modulating the MAPK/ERK and PI3 K/AKT/mTOR pathways; Inhibiting inflammation by suppression of NF-κB, AP-1 and NF-AT activity	[72]
		Maslinic acid	Anticancer activity	It can significantly suppress pancreatic tumor growth, induce tumor apoptosis and inhibit NF-κB-regulated anti-apoptotic gene expression	[73]
		Wulingzhic acid	Anticoagulative activity	Prolongation of thrombin time and inhibiting platelet aggregation	[13, 19]
		Wulingzhic acid A			
		Wulingzhic acid B			
		Trogopteroids A–G	Cytotoxic activity	Cytotoxicity effect against seven human tumor cells, such as HepG2, HL-60 and U937	[12]
		8β-hydroxy-3-oxopimara-15-ene			
		Akhdardiol			
		Isopimara-7(8),15-dien-3β-ol			
		Isopimara-8,15-dien-3β-ol			
		Sempervirol			
		Macrophynin E			
		Ferruginol	Gastroprotective activity	Increasing PGs content, protecting cells from lipid peroxidation and improving gastric ulcer healing	[74]
		Epifriedelanol	Antioxidant and anti-inflammatory activities	Attenuating the secondary injury in TBI rats by reducing serum cytokine levels and oxidative stress	[75]
	Flavonoids	Hinokiflavone	Anti-inflammatory activity	Inhibiting the LPS-induced expression of iNOS and COX-2 and the activation of NF-κB and ERK-1/2	[76]
		Amentoflavone	Anti-diabetic activity	Regulating glucose and lipid metabolism via anti-oxidant effects and activating the PI3K/Akt pathway	[77]
		Kaempferol-3-*O*-α-l-(2″*E*,4″*E*-di-*p*-coumaroyl)-rhamnoside	Anticoagulative activity	Significant prolongation of thrombin time	[14]
		Kaempferol-3-*O*-α-l-(3″*E*,4″*E*-di-*p*-coumaroyl)-rhamnoside			
		Kaempferol-3-*O*-α-l-(4″*E*-*p*-coumaroyl)-rhamnoside			
		Afzelin	Antioxidant and anti-inflammatory activities	Inhibiting particulate matter-induced proinflammatory cytokine mRNA expression and protein secretion; Inhibiting intracellular ROS generation, and p38 mitogen-activated protein kinase and transcription factor activator protein-1 component c-Fos and c-Jun activation	[78]
		Quercitrin	Gastroprotective and antioxidant activities	Inhibition of oxidative stress, regulation of mitochondrial dysfunction, and initiation of antioxidant defense	[79]
	Lignans	Trogopterins A, B, and C	Cytotoxic activity	Cytotoxicity effect against different types of cancer cells, such as HL-60, HeLa, and MCF-7	[16]
	Steroids	Daucosterol	Anti-colitis activity	Inhibiting dextran sulfate sodium (DSS)-induced colitis in mice by relieving inflammation and restoring the number of Treg cells	[80]
		β-sitosterol	Anti-inflammatory activity	Inhibition of intracellular adhesion molecule 1 expression in TNF-α-stimulated HAEC as well as the binding of U937 cells to TNF-α-stimulated HAEC and attenuating the phosphorylation of nuclear factor-kB p65	[81]
		Cholic acid	Anti-inflammatory activity	Significantly decreasing the content of PGE_2_ in inflammatory tissue	[82]
		Deoxycholic acid	Anti-inflammatory activity	Inhibiting fMLP-induced monocyte and neutrophil chemotaxis and calcium mobilization	[83]
		Ursodeoxycholic acid	Anti-inflammatory activity	Ameliorating experimental colonic inflammation in rats at a high dose (50 mg/kg day) by enhancing mucosal defense	[84]
		Taurocholic acid	Anti-inflammatory activity	Inhibiting the production of inflammatory mediators, such as NO, PGE_2_ and histamine	[85]
	Others	*Bis*(7-hydroxyheptyl)decanedioate	Anticoagulative activity	Significant prolongation of thrombin time	[17]
		*Bis*(7-hydroxyheptyl)octanedioate			
		Protocatechuic acid	Anti-inflammatory and analgesic activities	Significantly decreasing LPO, NO levels and increasing SOD, catalase and GSH levels; significantly increasing the hot pain threshold of experimental mice, and obviously decreasing the frequency of writhing body response	[91]
Hei-Bing-Pian	Steroids	Cholic acid	Anti-inflammatory activity	Significantly decreasing the content of PGE_2_ in inflammatory tissue	[82]
		Taurocholic acid	Anti-inflammatory activity	Inhibiting the production of inflammatory mediators, such as NO, PGE_2,_ and histamine	[85]

**Table 4:** Basic pharmacological data of commonly used fecal medicines mentioned in the article

Name	Type of extract	Animal or cell	N	Dose	Minimal active concentration	In vitro/In vivo	Positive control	Negative control	Duration	Effect and mechanism	Refs.
Wu-Ling-Zhi	Ethyl acetate extract	Rats	8	15–120 mg/kg	15 mg/kg	In vivo	Ranitidine	Normal saline	12 h	Inhibiting gastric acid secretion and protecting gastric mucosa	[86]
	Ethyl acetate extract	Rats	10	200–800 mg/kg	400 mg/kg	In vivo	Aspirin	Normal saline	30 min	Inhibiting the synthesis or release of prostaglandin E (PGE)	[25]
	Ethanol extract	MCF-7 cells	5	25–400 μg/ml	100 μg/ml	In vitro	–	DMSO	24 h	Increasing the expression levels of Caspase 3 and Caspase 9	[29]
	Ethyl acetate extract	Rabbits	3	0.62–1.04 mg/ml	0.78 mg/ml	In vitro	Aspirin	DMSO; 60% ethanol; PBS	10 min	Significant prolongation of thrombin time	[87]
	Water extract	Rabbits	5	30.8–616 μg/kg	56,225 μg/kg	In vitro	–	Normal saline	10–15 min	Significant increase of cAMP level in platelets	[88]
	Water extract	Rats	7	200–300 mg/kg	200 mg/kg	In vivo	-	Normal saline	20 min		
	Water extract	Rats	8	20–60 mg/kg	60 mg/kg	In vivo	Ligustrazine	Normal saline	7 days	Significant reduction of MDA and IL1-β levels; increasing SOD activity	[89]
	Water extract	Mice and rats	10	5–10 g/kg	5 g/kg	In vivo	Nimodipine	Normal saline	–	Reducing the brain water content, brain index and MDA level, and increasing SOD activity	[27]
	Water extract	Rats	15	2.5–10 g/kg	2.5 g/kg	In vivo	–	Normal saline	6 week	Down-regulation of the expression of intercellular adhesion molecule-1 in experimental atherosclerotic rats and reducing the degree of vascular endothelial lesions	[28]
Hei-Bing-Pian	Aqueous solution	Rats	8	0.5–2 g/kg	0.5 g/kg	In vivo	Hydrocortisone acetate	–	10 days	Increasing the expression of SOD and GSH, and reducing the expression of NO and MDA	[40]
	Aqueous solution	Rabbits	2	0.01–0.2 g/ml	–	In vitro	–	Tyrode’s solution	30 min	Regulating the cholinergic M receptor and histamine receptor	[41]
		Rats	6	0.05–0.1 g/kg	–	In vivo	–	–	30 min		
	Aqueous solution	Rats	8	1–5 g/kg	5 g/kg	In vivo	Domperidone	Normal saline	30 min	Accelerating the rate of gastric emptying, promoting gastrointestinal peristalsis and protecting gastric mucosa	[42]
		Mice	10	1–5 g/kg	5 g/kg	In vivo	Domperidone	Normal saline	30 min		
		Rats	8	1–5 g/kg	5 g/kg	In vivo	Kuai-Wei tablets	Normal saline	15 days		
	0.5% CMC-Na solution	Rats	20	4–12 g/kg	–	In vivo	–	Distilled water	12 w	No toxicity was observed after long-term administration	[43]

### Fecal medicines used in other traditional ethnic medicine systems in China

In addition to the TCM system, there are other traditional medical systems in China, such as Tibetan, Mongolian, Uygur, Tujia, Kazak, Yao, Korean, and Dai ethnic medicines. These ethnic medical systems have their own unique theories in the use of natural medicines. Therefore, it is also important to collect information about fecal medicines from these ethnic medical systems.

**Fig. 3:**
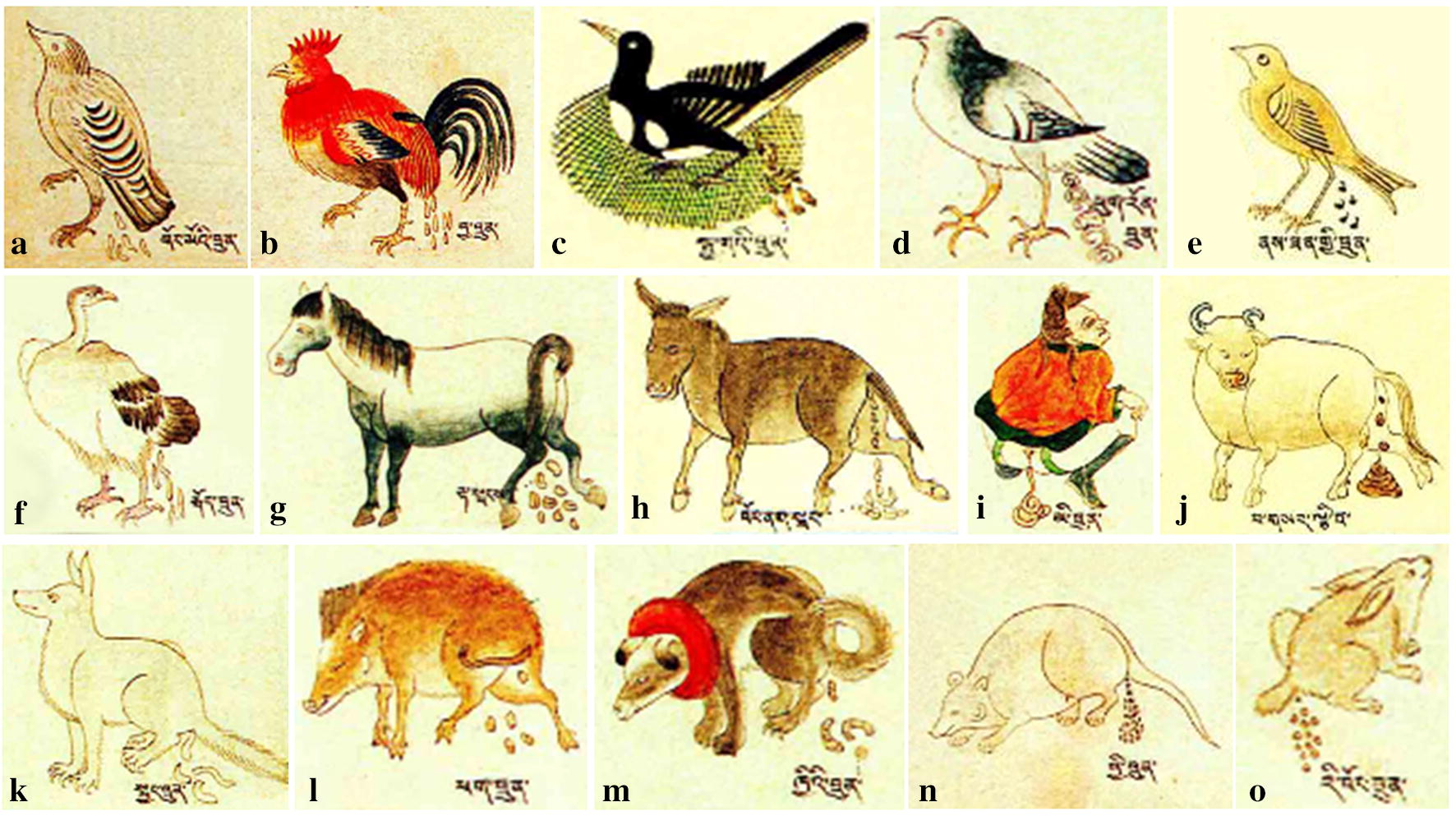
The Tibetan medical Thangka of “*The Four Medical Tantras*” vividly depicts some feces medicines and their origin animals (**a** *Tetrao urogallus*; **b** *Gallus gallus domesticus*; **c** *Pica pica*; **d** *Columba livia domestica*; **e** *Passer montanus*; **f** *Aegypius monachus*; **g** *Equus caballus orientalis*; **h** *Equus asinus*; **i** *Homo sapiens*; **j** *Bos taurus domesticus*; **k** *Canis lupus*; **l** *Sus scrofa*; **m** *Canis lupus familiaris*; **n** *Rattus rattus*; **o** *Lepus oiostolus*)

Traditional Tibetan medicine (TTM) is a representative ethnic medicine in China, and it has a unique fundamental theory, namely three elements theory consisting of “*rLung*”, “*mKhris*-*pa*” and “*Badkan*” [30]. In TTM system, the use of fecal medicines has a long history. The earliest Tibetan medicine monograph that recorded fecal medicines is “*The Four Medical Tantras*” [2]. Later, in the seventeenth century, famous “*Tibetan Medical Thangka of The Four Medical Tantras*” [31] was published by Sde-srid-sangs-rgyas-rgya-mtsho, which vividly depicted some commonly used fecal medicines in the form of wall chart (Fig. 3).

In this study, we found that the feces of 41 animals were used as medicines for the treatment of various diseases in 13 ethnic medical systems. More information on these medicines is provided in Table 2. Among them, the dry feces of *Gypaetus barbatus* or *Aegypius monachus* and the processed product of the feces of *Sus scrofa* are representative fecal medicines in Chinese ethnic medicine systems. Their traditional uses, chemical constituents and pharmacological activities have been described in detail in the following sections.

#### The dry feces of *Gypaetus barbatus* or *Aegypius monachus* (Jiu-Fen in Chinese)

The dry feces of *G. barbatus* or *A. monachus*, known as Jiu-Fen in Chinese, is a commonly used Tibetan medicine (Fig. 1d). It has the functions of strengthening stomach and promoting digestion. Jiu-Fen is used in the traditional Tibetan system of medicine for the treatment of dyspepsia, gastrointestinal dysfunction, gastric ulcer, and intestinal cancer in the past few decades [4, 6]. In addition, the “*Jing Zhu Materia Medica*” recorded that Jiu-Fen can be used to treat mental illness [4]. Nowadays, Jiu-Fen is frequently used in the clinical practice of TTM by combining other herbs. According to our statistics, there are 32 preparations containing Jiu-Fen in some monographs and drug standards of Tibetan medicine [6, 32–35]. The representative prescriptions include “Shi-Wei-Jiu-Fen Powder”, “Er-Shi-Jiu-Wei-Neng-Xiao Powder” and “Jian-Hua-Mu-Xiang Pills” (Table 5). The “*Tibetan Medicine Standards*” recorded that “Shi-Wei-Jiu-Fen Powder” can strengthen stomach and promote digestion [6]. Consequently, it is usually used to treat gastrointestinal diseases such as dyspepsia.

**Table 5:** Representative prescriptions containing fecal medicines recorded in official drug standards

Fecal medicine	Traditional medical system	The number of preparations	The name of prescriptions	Drug standard	Refs.
Wu-Ling-Zhi	TCM	15	Shi-Xiang-Zhi-Tong Pills; Tong-Jing Pills; Shao-Fu-Zhu-Yu Pills; Xiao-Jin Pills; Shi-Er-Wei-Yi-Shou Powder; Er-Shi-Wu-Wei-Song-Shi Pills; Qi-Wei-Tie-Xie Pills; Jiu-Qi-Niao-Tong Pills; Ping-Xiao Pills; Hua-Zheng-Hui-Sheng Tablets; Feng-Liao-Xing-Feng-Shi-Die-Da Wine; Yang-He-Jie-Ning Plaster; Tong-Jing-Bao Granules; Jie-Bai Pills; Bing-Lang-Si-Xiao Pills	Chinese Pharmacopoeia, 2015ed	[7]
Can-Sha	TCM	2	Shu-Jing-Huo-Luo Wine; Feng-Liao-Xing-Feng-Shi-Die-Da Wine	Chinese Pharmacopoeia, 2015ed	[7]
Ye-Ming-Sha	TCM	1	Huang-Lian-Yang-Gan Pills	Chinese Pharmacopoeia, 2015ed	[7]
Jiu-Fen	Tibetan EM	12	Shi-Wei-Jiu-Fen Powder; Er-Shi-Jiu-Wei-Neng-Xiao Powder; Jian-Hua-Mu-Xiang Pills; Shi-San-Wei-Mu-Xiang Pills; Shi-San-Wei-Shi-Liu Pills; De-Ma-Shi-San-Wei-Shi-Liu Pills; Shi-San-Wei-Mu-Xiang Powder; Jia-Wei-Bai-Yao Pills; Qu-Hui Pills; Qu-Han-Quan-Lü Powder; Song-Shi-Da-Peng Pills; Shi-San-Wei-Qing-Lan Pills	Drug Standards of Tibetan Medicines; Tibetan Medicine Standards	[6, 35]
Hei-Bing-Pian	Tibetan EM	14	Shi-Wei-Hei-Bing-Pian Powder; Shi-Yi-Wei-Jin-Se Pills; Shi-Wu-Wei-Zhi-Xie-Mu Powder; Gan-Lu-Ling Pills; Er-shi-Si-Wei-A-Wei Powder; Shi-Yi-Wei-He-Zi Powder; Shi-San-Wei-Di-Da Powder; Niu-Huang-Qing-Peng Pills; He-Zi-Neng-Xiao Pills; He-Zi-Qing Peng-Pills; Jin-Se-Di-Da Pills; Er-shi-Jiu-Wei-Qiang-Huo Powder; Shi-San-Wei-Shu-Tu-Qing-Peng Powder; Shi-Yi-Wei-Jin-Se Powder	Drug Standards of Tibetan Medicines; Tibetan Medicine Standards	[6, 35]
Wu-Ling-Zhi	Mongolian EM	23	Yun-Xiang-Shi-Wu-Wei Pills; Zhi-Li-Shi-Wei-Hei Pills; Niu-Huang-Shi-San-Wei Pills; Wen-Guan-Mu-Shi-Wei Powder; Ji-Xiang-An-Kun Pills; Li-Gan-He-Wei Pills; He-Zi-Wu-Wei Capsules; Bu-Sheng-Jian-Wei-Er-Shi-Yi Pills; Feng-Xiang-Zhi-Shi-Wei Pills; Cao-Guo-Jian-Pi Pills; Ha-Dun-Hai-Lu-Mu-Le-Shi-San-Wei Pills; Ha-Dun-Hai-Lu-Mu-Le-Jiu-Wei Pills; Jian-Wei-Shi-Wei Pills; Jian-Pi-Wu-Wei-Pills; Qing-Gan-Qi-Wei Powder; Tiao-Yuan-Da-Bu-Er-Shi-Wu-Wei Decoction; Ju-Hua-Qi-Wei Capsules; Qing-Gan-Jiu-Wei Powder; Qing-Shen-Re-Shi-Wei Powder; Qing-Wen-Shi-Er-Wei Pills; Qing-Wen-Zhi-Tong-Shi-Si-Wei Pills; Qing-Wen -Li-Dan-Shi-San-Wei Pills; Xi-Hong-Hua-Shi-Liu-Wei Powder	Drug Standards of Mongolian Medicines	[90]
Hei-Bing-Pian	Mongolian EM	7	Zhi-Li-Shi-Wu-Wei Pills; He-Zi-Wu-Wei Capsules; A-Na-Ri-Ba-Wei Powder; Ha-Ri-Shi-Er-Wei Powder; Ha-Dun-Hai-Lu-Mu-Le-Shi-San-Wei Pills; Qing-Gan-Er-Shi-Qi-Wei Pills; Shi-Wei-Hei-Bing-Pian Powder	Drug Standards of Mongolian Medicines	[90]
Ye-Ming-Sha	Mongolian EM	1	Ming-Mu-Shi-Liu Pills	Drug Standards of Mongolian Medicines	[90]
Long-Xian-Xiang	Uygur EM	1	Yi-Mu-Sa-Ke Tablets	Drug Standards of Uygur Medicines	[92]

The use of Jiu-Fen in the traditional Tibetan system of medicine has a long history, but modern research on the chemical composition, quality control and pharmacodynamic evaluation of Jiu-Fen has not yet been carried out. Therefore, further studies are needed to prove its medicinal values in gastrointestinal diseases treatment, identify active compounds and elucidate the underlying mechanisms with the help of modern chemistry and pharmacology methods.

#### The processed product of the feces of *Sus scrofa* (Hei-Bing-Pian in Chinese)

The processed product of the feces of *Sus scrofa* (wild boar), known as Hei-Bing-Pian (Chinese name), is a widely used Tibetan and Mongolian medicine in China (Fig. 1e). Its processing method was recorded in the “*Chinese Materia Medica for Tibetan Medicine*”: firstly, the dry feces of *Sus scrofa* is put into a ceramic jar, and yellow mud (adding a small amount of salt) is used to seal the ceramic jar. Secondly, the ceramic jar is calcined with fire until it turns gray outside. Then, the black matter is taken out from the ceramic jar, which is Hei-Bing-Pian [36]. In traditional Tibetan system of medicine, Hei-Bing-Pian is described as pungent in flavor and hot in nature. It is commonly used for the treatment of dyspepsia, gallbladder diseases, stomachache, and plague [6]. According to our statistics, there are 14 Tibetan medicine preparations containing Hei-Bing-Pian in official drug standards. The representative prescriptions include “Shi-Wei-Hei-Bing-Pian Powder”, “Shi-Yi-Wei-Jin-Se Pills” and “Shi-Wu-Wei-Zhi-Xie-Mu Powder” (Table 5). The “*Drug Standards of Tibetan Medicines*” recorded that “Shi-Wei-Hei-Bing-Pian Pills” is usually used to treat stomach and gallbladder diseases, such as dyspepsia, anorexia, jaundice, gallstones and nausea [35].

It has been reported that Hei-Bing-Pian contains a variety of inorganic elements, such as Fe, Ca, Zn, K, Cu, Mn, Co, Ti, and Mg. At present, the contents of these elements in Hei-Bing-Pian have been determined by using atomic absorption spectrometry or spectrophotometry [37, 38]. The elements with high levels are Ca (18,570 μg/g), K (11,625 μg/g), Mg (9975 μg/g), and Fe (7800 μg/g). Furthermore, two bile acids (i.e., cholic acid and taurocholic acid) were detected and quantified in Hei-Bing-Pian by using ultra high-performance liquid chromatography-mass spectrometry method [21]. Besides, Chang et al. [39] developed a spectrophotometric method to determine the absorption force of Hei-Bing-Pian on tartrazine, and used this adsorption force as an indicator to control the quality of Hei-Bing-Pian.

Modern pharmacological study has demonstrated that Hei-Bing-Pian can prevent mucosal damage caused by experimental colitis in rats. Compared with the model group, the high and low doses of Hei-Bing-Pian can significantly reduce the damage of colonic mucosa congestion, hyperplasia and ulcer (*p* < 0.05), and significantly increase the levels of superoxide dismutase (SOD) and glutathione (GSH) [40]. Cai et al. [41] reported the effect of Hei-Bing-Pian on the intestinal smooth muscle function of animals. It was found that Hei-Bing-Pian had no obvious effect on normal isolated ileum, and could not antagonize the inhibitory effect of atropine, adrenaline and promethazine on isolated ileum smooth muscle. However, it could significantly inhibit histamine-induced ileal smooth muscle excitation, and the inhibition rate was 25%. Moreover, in vivo studies showed that Hei-Bing-Pian could inhibit the contraction effect of pilocarpine on ileal smooth muscle. These results indicate that the effect of Hei-Bing-Pian on intestinal smooth muscle is related to the cholinergic M receptor and histamine receptor. Bai et al. [42] found that Hei-Bing-Pian could significantly accelerate gastric emptying in rats and promote the propulsive speed of activated carbon in the small intestine of mice. Moreover, the high dose of Hei-Bing-Pian could significantly promote the healing of chronic gastritis caused by acetic acid, and had obvious protective effect on gastric mucosal injury induced by cold stress. Besides, in order to make better use of Hei-Bing-Pian, its long-term toxic effects have been studied by Li et al. The results showed that, after 12 weeks of administration of Hei-Bing-Pian, there were no significant changes in body weight, blood biochemical parameters, histopathology, and several organ indexes (e.g., heart, liver, spleen, kidney, and thymus) in rats, compared with the control group, which indicated that Hei-Bing-Pian has no potential toxicity [43]. The basic pharmacological data of Hei-Bing-Pian and its ingredients are shown in Tables 3 and 4.

### Similarities and differences of fecal medicines related to treated diseases in Chinese traditional medical systems

Every traditional medical system in China has its own unique theory or medication experience. Therefore, the same fecal medicines are used in different medical systems, and their therapeutic uses may be different. A detailed comparison of these differences would help researchers and traditional medical practitioners to better understand the indications of fecal medicines and promote their development and utilization. In this study, we compared the similarities and differences of therapeutic uses of Wu-Ling-Zhi and Hei-Bing-Pian in different traditional medical systems, including TCM, Tibetan EM, Korean EM, Dai EM, Yao EM, Tujia EM, Nu EM, and Mongolian EM. Additional details are provided in Table 6. The results indicate that Wu-Ling-Zhi is commonly used to treat amenorrhea and dysmenorrhea in most traditional medical systems. However, its therapeutic uses also have some obvious differences in different medical systems. For example, in the Tibetan EM, Wu-Ling-Zhi can be used to treat stomachache, whereas in the Mongolian EM, it is mainly used to treat diarrhea, gout and itching. Moreover, Wu-Ling-Zhi can treat cold, whooping cough and fever in the Nu EM. Hei-Bing-Pian has the same therapeutic use in TCM and Tibetan EM systems. It is widely used in both systems to treat dyspepsia, biliary diseases, plague and distending pain in the stomach. There is no difference in the therapeutic use of Hei-Bing-Pian in the two medical systems.

**Table 6:** Similarities and differences of fecal medicines related to treated diseases in Chinese traditional medical systems

Name	Traditional medical system	Original species	Identical indications	Different indications	Refs.
Wu-Ling-Zhi	TCM	*Trogopterus xanthipes* Milne-Edwards	Amenorrhea and dysmenorrhea	Stabbing pain in the chest and abdomen, swelling and aching due to traumatic injury, and snake bites (external use)	[5, 8, 52]
	Tibetan EM			Stomachache	[59, 60]
	Korean EM			Stabbing pain in chest and abdomen	[60]
	Dai EM			Snake bites (external use)	[60]
	Yao EM			Epilepsy	[63]
	Tujia EM			Swelling and aching due to traumatic injury, and snake bites (external use)	[60]
	Nu EM		–	Cold, whooping cough and fever	[60]
	Mongolian EM		–	Diarrhea, gout and itching	[62]
Hei-Bing-Pian	Tibetan EM	*Sus scrofa* L.	Dyspepsia, biliary diseases, plague and distending pain in the stomach	–	[6, 36, 59]
	Mongolian EM				

## Conclusion and future perspectives

Chinese traditional medicine is an important part of the world’s medical system. In long-term clinical practice, ancient Chinese doctors have accumulated invaluable experience in the use of fecal medicines. As shown in Tables 1 and 2, some fecal medicines have been found to be effective in treating amenorrhea, dysmenorrhea dyspepsia, diarrhea, fever, and stomachache. These traditional medication knowledge are valuable assets. Currently, some fecal medicines (e.g., Wu-Ling-Zhi, Jiu-Fen and Hei-Bing-Pian) are still used in clinical practice. A total of 76 preparations containing fecal medicines were recorded in the latest official drug standards (Table 5). Extensive clinical application demonstrates the role and value of fecal medicines in Chinese medical systems. Moreover, Wu-Ling-Zhi extracts and its chemical constituents have been proved to possess a wide spectrum of biological activities, such as anti-inflammatory, anticoagulative and antioxidant effects (Tables 3 and 4). Some possible molecular mechanisms have also been revealed. The results of these modern pharmacological studies provide some evidences to prove the scientific nature of fecal medicines. However, most fecal medicines still lack experimental evidences. For example, Jiu-Fen is a commonly used Tibetan medicine. However, so far, no biological activities or active ingredients have been reported for this drug. Therefore, in order to better develop and utilize these fecal medicines, more in vivo pharmacological studies and even clinical evaluations should be performed to prove their scientific and medicinal value.

Fortunately, an in-depth study of gut microbiota has provided an opportunity to interpret the scientific connotation of some traditional fecal medicines, such as the yellow soup. This soup is a fresh fecal suspension of *Homo sapiens* commonly used to treat food poisoning, severe diarrhea, heat toxins, and unconsciousness due to high fever. Zhang et al. [44, 45] believe that the efficacy of yellow soup is mainly caused by the gut microbiota from fresh fecal water, and its principle for treating diseases is similar to the fecal microbiota transplantation method of modern medicine. Therefore, reconstructing the gut microbiota of patients may be the mechanism of action of fresh fecal medicines for treating diseases.

However, most fecal medicines are derived from dry feces (e.g., Wu-Ling-Zhi) or processed products (e.g., Hei-Bing-Pian). During drying and processing, these fecal medicines lose the living microbiota. Therefore, their mechanisms for treating diseases may be different from fresh feces. Feces are intestinal excretions of humans or animals. The chemical constituents in feces are mainly derived from the host or dietary metabolites. These metabolites may be the key active constituents of fecal medicinal materials. For example, the terpenoids, flavonoids and lignans contained in Wu-Ling-Zhi are closely related to the foods eaten by *Trogopterus xanthipes* (e.g., *Platycladus orientalis* leaves, *Pinus tabulaeformis* bark, and peach kernels). These diet-derived metabolites may be the pharmacologically active ingredients of Wu-Ling-Zhi. Moreover, some bile acids (e.g., deoxycholic acid, lithocholic acid and taurocholic acid) have been found in Wu-Ling-Zhi and Hei-Bing-Pian [21]. These bile acids are the final metabolites of cholesterol under the common metabolism of liver and gut microbiota. Bile acids are endocrine-signaling molecules that regulate metabolic processes, including glucose, lipid and energy homeostasis, by regulating gut microbiota or activating bile acid receptors, such as the farnesoid X receptor (FXR) and G protein-coupled bile acid receptor 1 [46–48]. Distrutti et al. [49] reported that bile acids-activated receptors are targets for maintaining intestinal integrity. Gadaleta et al. [50] found that FXR activation could prevent chemically induced intestinal inflammation with an improvement of colitis symptoms and inhibition of epithelial permeability. In addition, bile acids can also regulate cardiovascular functions via receptor-dependent and -independent mechanisms [51]. These findings provide a rationale to explore the mechanisms of Hei-Bing-Pian and Wu-Ling-Zhi in the treatment of gastrointestinal and cardiovascular diseases, respectively.

With the continuous development of science and technology, some unique but sometimes incomprehensible drugs in traditional medical systems will gradually be recognized. In this study, we provide the first comprehensive data compilation of fecal medicines used in Chinese traditional medical systems. The information recorded in ancient monographs and drug standards, such as original species, traditional uses and indications, can provide a good reference for the development and utilization of fecal medicines. In view of the current research status of fecal medicines, future research may focus on the following aspects: (1) applying multidisciplinary methods to further prove their effectiveness and medicinal value, (2) revealing their active ingredients associated with clinical efficacy using phytochemical and pharmacodynamic methods, and (3) elucidating the mechanisms of action of fecal medicines based on gut microbiota or receptor-mediated signaling pathways.

## Data Availability

The datasets used and/or analysed during the current study are available from the corresponding author on reasonable request.

## Abbreviations

TCM: traditional Chinese medicine
TTM: traditional Tibetan medicine
EM: ethnic medicine
